# Progressive induction of hepatocyte progenitor cells in chronically injured liver

**DOI:** 10.1038/srep39990

**Published:** 2017-01-04

**Authors:** Naoki Tanimizu, Norihisa Ichinohe, Masahiro Yamamoto, Haruhiko Akiyama, Yuji Nishikawa, Toshihiro Mitaka

**Affiliations:** 1 Department of Tissue Development and Regeneration, Research Institute for Frontier Medicine, Sapporo Medical University School of Medicine, S-1, W-17, Chuo-ku, Sapporo 060-8556, Japan; 2 Division of Tumor Pathology, Department of Pathology, Asahikawa Medical University, 1-1-1 Higashi 2 jou, Midorigaoka, Asahikawa 078-1580, Japan; 3 Department of Orthopedics, Gifu University School of Medicine, Yanagido 1-1, Gifu 501-1194, Japan

## Abstract

Differentiated epithelial cells show substantial lineage plasticity upon severe tissue injuries. In chronically injured mouse livers, part of hepatocytes become Sry-HMG box containing 9 (Sox9) (+) epithelial cell adhesion molecule (−) hepatocyte nuclear factor 4 α (+) biphenotypic hepatocytes. However, it is not clear whether all Sox9^+^ hepatocytes uniformly possess cellular properties as hepatocyte progenitors. Here, we examined the microarray data comparing Sox9^+^ hepatocytes with mature hepatocytes and identified CD24 as a novel marker for biphenotypic hepatocytes. Immunohistochemical analyses showed that part of Sox9^+^ hepatocytes near expanded ductular structures expressed CD24 in the liver injured by 3,5-diethoxycarbonyl-1,4-dihydro-collidine (DDC) diet and by bile duct ligation. Indeed, Sox9^+^ hepatocytes could be separated into CD24^−^ and CD24^+^ cells by fluorescence activated cell sorting. The ratio of CD24^+^ cells against CD24^−^ ones in Sox9^+^ hepatocytes gradually increased while DDC-injury progressed and colony-forming capability mostly attributed to CD24^+^ cells. Although hepatocyte markers were remarkably downregulated in of Sox9^+^ CD24^+^ hepatocytes, they re-differentiated into mature hepatocytes *in vitro* and *in vivo*. Our current results demonstrate that the emergence of biphenotypic hepatocytes is a sequential event including the transition from CD24^−^ and CD24^+^ status, which may be a crucial step for hepatocytes to acquire progenitor properties.

It has been considered that many types of epithelial tissues and organs contain tissue specific stem/progenitor cells, which continuously supply multiple types of cells throughout life. However, except the gastrointestinal tracts and the skin^1^,^2^, it remains ambiguous whether tissue specific stem/progenitor cells majorly contribute to tissue/organ homeostasis and regeneration.

Liver stem/progenitor cells (LPCs) are defined as bipotential cells differentiating into two types of liver epithelial cells, hepatocytes and cholangiocytes. Hepatoblasts, embryonic LPCs, which can be identified as Dlk-1^+^, CD13^+^, E-cadherin^+^, and Liv-2^+^ cells in mid gestation^3^,^4^,^5^,^6^,^7^, and differentiate into hepatocytes and cholangiocytes during development^8^. Adult LPCs are enriched in cellular fractions positive for epithelial cell adhesion molecule (EpCAM), CD13, CD133, and MIC1–1C3 isolated from healthy and injured livers^9^,^10^,^11^,^12^. Furthermore, recent works using a genetic lineage tracing technique showed that LPCs marked with Sry-HMG box containing 9 (Sox9) expression continuously supplied new hepatocytes in developing liver^13^ and in healthy adult one^14^, whereas LPCs labeled with osteopontin (OPN) expression differentiated to hepatocytes during the recovery from chronic injury induced by choline-deficient ethionine-supplemented (CDE) diet^15^. However, more recently, a possibility has been proposed that mature hepatocytes (MHs) rather than LPCs mainly contribute to supplying new MHs in healthy and in injured livers^16^,^17^,^18^. We previously demonstrated that part of MHs show intermediate status between hepatocytes and cholangiocytes in chronically injured livers and behave as hepatocyte progenitors^19^. These intermediate cells were called “biphenotypic hepatocytes”^20^. However, it remains largely unknown whether biphenotypic hepatocytes derived from MHs are homogeneous population and how the lineage plasticity of MHs is controlled.

We performed microarray analysis comparing Sox9^+^EpCAM^−^ biphenotypic hepatocytes isolated from 3,5-diethoxycarbonyl-1,4-dihydro-collidine (DDC)-injured livers with MHs. Based on the result, we separated Sox9^+^EpCAM^−^ hepatocyte progenitors into CD24^−^ and CD24^+^ populations. The ratio of CD24^+^ cells against CD24^−^ ones gradually increased during DDC-injury and the capability of clonal proliferation mostly attributed to CD24^+^ cells. Even though CD24^+^ cells remarkably reduced most of hepatocyte markers, they re-differentiated into MHs both *in vitro* and *in vivo*. In contrast, their potential differentiating into cholangiocyte-like cells was limited. Thus, part of MHs acquire clonal proliferative capability, through the transition from CD24^−^ to CD24^+^ status, whereas they maintain potential re-differentiating into MHs. It may be important that biphenotypic hepatocytes maintain the potential re-differentiating into MHs for liver tissue regeneration.

## Results

### CD24 is upregulated in Sox9^+^EpCAM^−^ hepatocyte progenitors

As previously reported, biphenotypic hepatocytes induced in DDC-, bile duct ligation (BDL)-, and CDE-injured livers can be identified as Sox9^+^EpCAM^−^ cells^19^. The genetic lineage tracing using Mx1Cre:ROSA26, in which MHs were labeled with LacZ after poly(I:C) injection, demonstrated that Sox9^+^ biphenotypic hepatocytes are derived from MHs. In this work, we performed another genetic lineage tracing with ROSA26 mice and AAV8-TBG-Cre, in which MHs were labeled with LacZ after injection of AAV8 into mice, and confirmed that Sox9^+^ biphenotypic cells were originated from MHs (Fig. S1).

**Figure 1:**
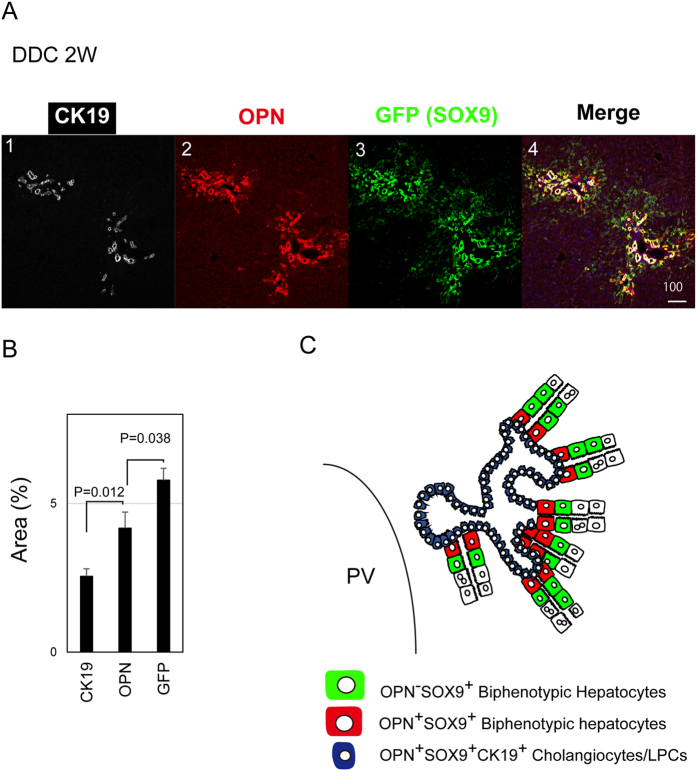
Biphenotypic hepatocytes induced by chronically liver injury consist of heterogeneous cell populations. (**A**) Expression of SOX9, CK19, and OPN in DDC-injured liver. SOX9^+^OPN^+^ hepatocytes are evident around expanded CK19^+^ ductular cells in DDC-injured liver. SOX9^+^OPN^−^ hepatocytes are more distant from ductular structures than SOX9^+^OPN^+^ hepatocytes. A liver section prepared from Sox9-EGFP mice fed with DDC-diet for 2 weeks was stained with anti-GFP, anti-OPN, and anti-CK19 antibodies. A bar represents 100 μm. (**B**) Quantitative analysis for expression of CK19, SOX9, and OPN. Liver sections were prepared from 3 Sox9-EGFP mice fed with DDC-diet for 2 weeks. Three images were selected in each section and the percentage of CK19^+^, SOX9^+^, and OPN^+^ areas were quantitated on ImageJ. (**C**) Schematic view for the localization of SOX9^+^OPN^+^ and SOX9^+^OPN^−^ hepatocytes. SOX9^+^OPN^+^ hepatocytes are localized near CK19^+^SOX9^+^OPN^+^ ductular structures. SOX9^+^OPN^−^ hepatocytes exist outside the region where SOX9^+^OPN^+^ ones are localized.

**Figure 2:**
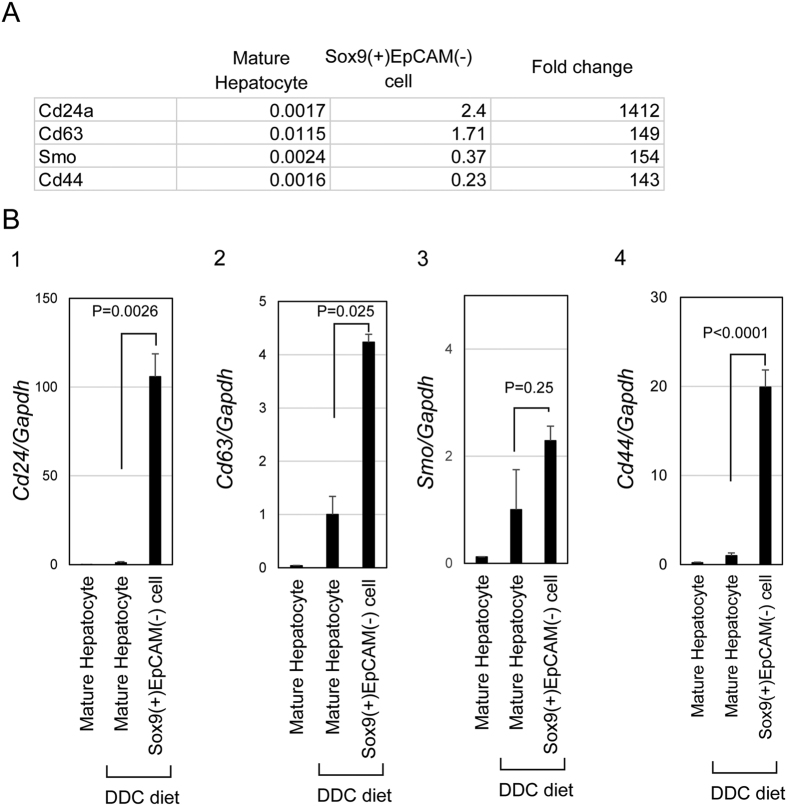
CD24 is upregulated in Sox9^+^EpCAM^−^ biphenotypic hepatocytes. (**A**) A list of surface markers upregulated in Sox9^+^EpCAM^−^ biphenotypic hepatocytes as compared with MHs. *Cd24, Cd63 Smoothened (Smo)*, and *Cd44*, are suggested to be increased in Sox9^+^EpCAM^−^ cells as compared with MHs. Microarray analysis comparing Sox9^+^EpCAM^−^ biphenotypic hepatocytes isolated from DDC-injured livers with MHs was performed. (**B**) Quantitative analysis for expression of *Cd24, Cd63*, and *Smo and Cd44. Cd24* is remarkably upregulated in Sox9^+^EpCAM^−^ biphenotypic hepatocytes as compared with MHs isolated from healthy and DDC-injured livers. *Cd63, Smoothened (Smo), and CD44* are also upregulated in Sox9^+^ progenitors, though the increase of these genes are not prominent as compared with *Cd24*. Sox9^+^EpCAM^−^ cells and MHs were isolated from livers of Sox9-EGFP mice fed with DDC-diet for 2 weeks. MHs isolated from a healthy liver were also used for analyses. Cell isolation was performed 3 times, independently. Relative expression levels against that in MHs isolated from DDC-injured livers are shown in graphs. Error bars represent SEM.

In addition to Sox9, part of hepatocytes also expressed osteopontin (OPN) in chronically injured livers^20^. However, we found that the OPN expression was restricted in Sox9^+^ biphenotypic hepatocytes near EpCAM^+^ expanding ductules (Fig. 1). This result suggested that Sox9^+^ biphenotypic hepatocytes consist of heterogeneous cell populations. In order to further clarify such heterogeneity of biphenotypic hepatocytes, we compared gene expression profile of Sox9^+^EpCAM^−^ cells isolated from DDC-injured livers with that of MHs isolated from the healthy livers by microarray (GEO accession number: GSE78778). Among cell surface molecules, *Cd24, Cd*63, *Smoothened (Smo*), and *Cd44* were upregulated in Sox9^+^EpCAM^−^ cells (Fig. 2A). We further examined their expressions in Sox9^+^EpCAM^−^ cells and MHs by quantitative PCR, and confirmed that *Cd24* was upregulated by 100-fold in Sox9^+^EpCAM^−^ biphenotypic hepatocytes as compared with MHs (Fig. 2B). This result suggests that CD24 is a new marker for biphenotypic hepatocytes.

### Proliferative hepatocyte progenitors are enriched in Sox9^+^EpCAM^−^CD24^+^ fraction

**Figure 3:**
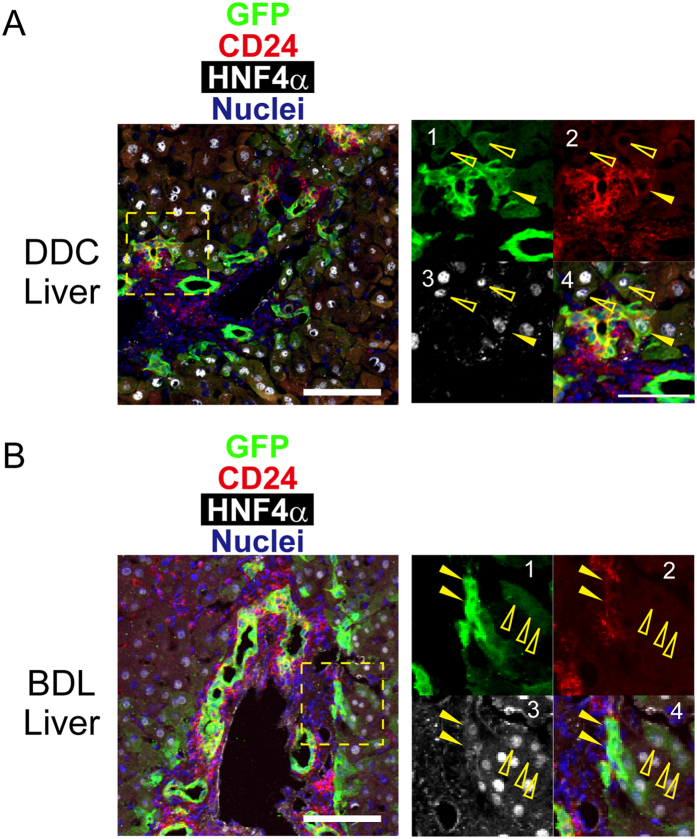
CD24 expression in DDC and BDL-injured livers. (**A**) CD24 is expressed in part of Sox9^+^ biphenotypic hepatocytes in DDC injured livers. Most of Sox9^+^HNF4α^−^ ductular cells are CD24^+^, whereas Sox9^+^HNF4α^+^ biphenotypic hepatocytes are CD24^+^ (closed arrowheads) and CD24^−^ (open arrowheads). Sox9^+^HNF4α^+^CD24^+^ cells are preferentially localized at the boundary between ductular structure and hepatic cord in this injury model. Ductular reaction was induced by feeding Sox9-EGFP mice with DDC diet for 2 weeks. Scale bars represent 50 μm. (**B**) CD24 is expressed in part of Sox9^+^ biphenotypic hepatocytes in BDL livers. Similar to DDC injured livers, Sox9^+^HNF4α^+^ biphenotypic hepatocytes are partly CD24^+^ (closed arrowheads) and CD24^−^ (open arrowheads). Ductular reaction was induced by tying the common bile duct and liver tissue was examined 2 weeks later. Scale bars represent 50 μm.

We tested a possibility that CD24 might recognize a subpopulation of Sox9^+^ biphenotypic hepatocyte. We first examined expression of CD24 in liver tissues injured by DDC-diet or BDL (Fig. 3). In this analysis, we could identify expanding ductular cells and biphenotypic hepatocytes as GFP^+^ hepatocyte nuclear factor 4 α (HNF4α)^−^ and GFP^+^HNF4α^+^ cells, respectively. Immunofluorescence analysis indicated that most of ductular Sox9^+^ cells were positive for CD24. On the other hand, part of Sox9^+^HNF4α^+^ biphenotypic hepatocytes were CD24^+^ in the boundaries between expanded ductular structures and hepatic cords (**closed arrowheads in panels 1~4 of** Fig. 3A,B), whereas Sox9^+^HNF4α^+^ biphenotypic hepatocytes apart from ductular structures were CD24^−^ (**open arrowheads in panels 1~4 of** Fig. 3A,B). The ratios of Sox9^+^HNF4α^+^CD24^+^/Sox9^+^HNF4α^+^CD24^−^ biphenotypic hepatocytes were 7.2/64.0 cells/mm^2^ and 14.4/106.4 cells/mm^2^ in DDC-injured and in BDL-injured livers, respectively (Table S1).

**Figure 4:**
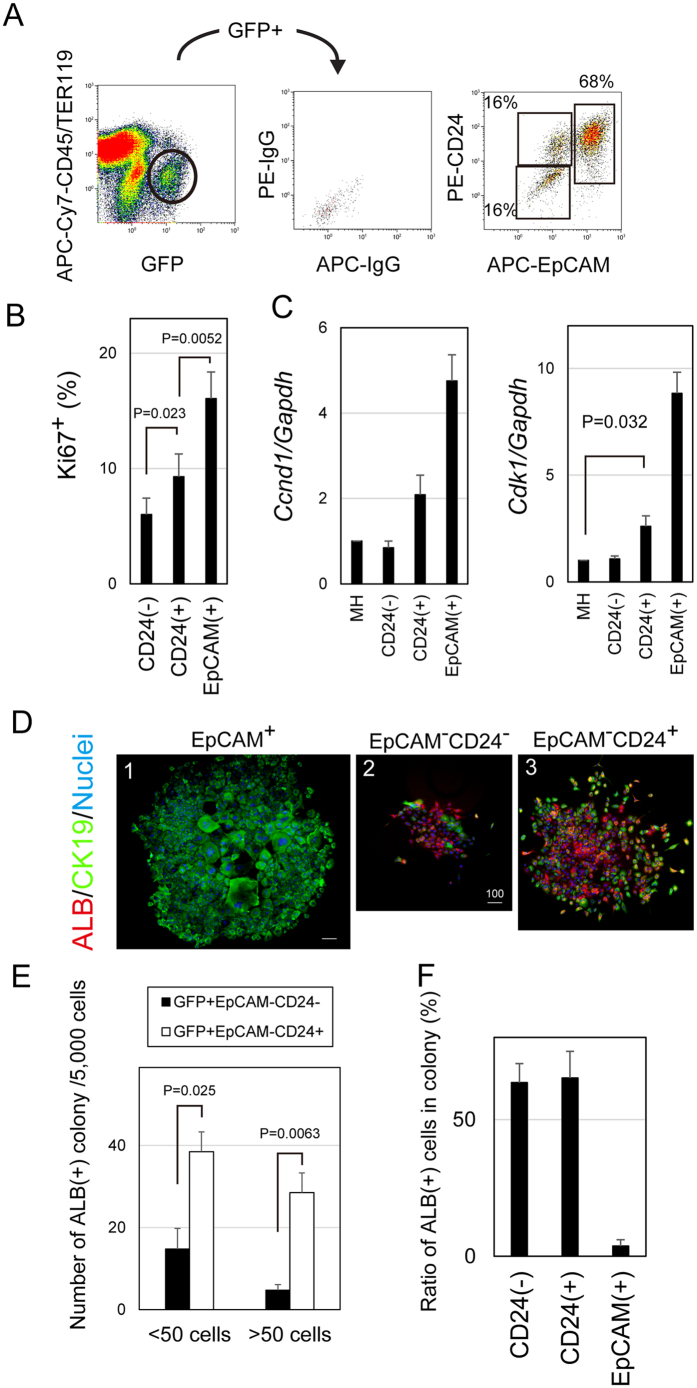
Sox9^+^EpCAM^−^ biphenotypic hepatocytes are separated into CD24^−^ and CD24^+^ cells. (**A**) FACS analysis shows that Sox9^+^EpCAM^−^ fraction can be separated into CD24^−^ and CD24^+^ cells. Cells were isolated from livers of Sox9-EGFP mice fed with DDC-diet for 2 weeks and Sox9^+^EpCAM^−^ cells were analyzed for CD24 expression. (**B**) CD24^+^ cells show higher proliferative potential as compared with CD24^−^ ones. CD24^+^ fraction contains more Ki67^+^ cells than CD24^−^ one. Cell isolation from mice fed with DDC diet for 2 weeks was repeated for 3 times independently. Smear samples were prepared with a Cytospin and stained with anti-mouse Ki67 antibody. Error bars represent SEM. (**C**) Expression of cell cycle related genes in biphenotypic hepatocytes. CD24^+^ cells express *Ccnd1* and *Cdk1* more than CD24^−^ ones. Notably, CD24^+^ cells, but not CD24^−^ ones, express *Cdk1* at significantly higher level as compared with MHs. Error bars represent SEM. (**D**) ALB^+^ large bipotential colonies emerge from CD24^+^ cells. Colonies containing ALB^+^ hepatocytes are formed both from GFP^+^EpCAM^−^CD24^−^ and GFP^+^EpCAM^−^CD24^+^ cells. However, a bipotential colony derived from a CD24^+^ cell is apparently larger than that from a CD24^−^ one (**panels 2&3**). In contrast, colonies emerged from EpCAM^+^ cells are mostly consist of only CK19^+^ cells (**panel 1**). At day 7 of culture, cells were fixed and stained with anti-ALB and anti-CK19 antibodies. Scale bars represent 50 μm. (**E**) CD24^+^ cells form larger colonies. The graph shows the number of ALB^+^ colonies emerged in colony assay. After staining cells with anti-ALB, anti-CK19, and Hoechst 33324, the number of cells in each colony and the number of colonies were counted. Colony assay was repeated 4 times and the average values of the colony number are shown in the graph. Error bars represent SEM. (**F**) Colonies derived from CD24^−^ and CD24^+^ cells similarly contain ALB^+^ cells. ALB^+^ cells represent more than 50% of cells in colonies derived from EpCAM^−^CD24^−^ and EpCAM^−^CD24^+^ cells. In contrast, only about 4% of cells are ALB^+^ in colonies from EpCAM^+^ cells. Culture was repeated 3 times, independently. The ratio of ALB^+^ cells were evaluated after staining with anti-ALB and CK19 antibodies.

Immunohistochemical data demonstrated that Sox9^+^ hepatocytes emerged in chronically injured livers consist of CD24^−^ and CD24^+^ cells. Indeed, fluorescence activated cell sorting (FACS) analysis for cells isolated from Sox9-EGFP mice fed with DDC-diet demonstrated that Sox9^+^EpCAM^−^ cells could be separated into CD24^−^ and CD24^+^ ones (Fig. 4A). FACS analysis also showed that the ratio of CD24^+^ cells against CD24^−^ ones gradually increased as a period of DDC-feeding was extended (Fig. S2A). We also found that Sox9^+^EpCAM^−^CD24^−^ cells became Sox9^+^EpCAM^−^CD24^+^ ones during 2 weeks of culture, which did not appear in culture of EpCAM^+^ cells (Fig. S2B). Genetic lineage tracing with ROSA26 mice and AAV8-TBG-Cre showed that CD24^+^ hepatocytes were originated from MHs (Fig. S2C). These results indicate that Sox9^+^EpCAM^−^CD24^+^ cells are derived from MHs via Sox9^+^EpCAM^−^CD24^−^ cells.

Then, we isolated Sox9^+^EpCAM^−^CD24^−^ and Sox9^+^EpCAM^−^CD24^+^ cells from DDC-injured livers and examined their proliferative capability. The CD24^+^ fraction contained Ki67^+^ cells more than the CD24^−^ one (Fig. 4B). Furthermore, CD24^+^ cells expressed *cyclin D1 (Ccnd1*) and *cyclin dependent kinase 1 (Cdk1*) more than CD24^−^ ones (Fig. 4C). *Cdk 1* expression in CD24^+^ cells but not CD24^−^ ones was expressed at significantly higher level as compared with MHs. These results suggest that CD24^+^ cells possess higher proliferative capability as compared with CD24^−^ ones. To confirm this assumption, we performed a clonal culture of CD24^−^ and CD24^+^ cells. In this culture condition, EpCAM^+^ cells form large colonies consisting of ALB^−^CK19^+^ cholangiocyte-like cells (Fig. 4D-1). Although colonies containing ALB^+^ hepatocytes and CK19^+^ cholangiocytes emerged in both cultures (Fig. 4D-2 & 3), Sox9^+^EpCAM^−^CD24^+^ cells formed more and larger colonies than Sox9^+^EpCAM^−^CD24^−^ ones (Fig. 4E). The ratio of ALB^+^ cells were similar in colonies derived from Sox9^+^EpCAM^−^CD24^−^ and Sox9^+^EpCAM^−^CD24^+^ (Fig. 4F). These results indicate that biphenotypic hepatocytes with higher proliferative potential are enriched in Sox9^+^EpCAM^−^CD24^+^ fraction.

### Sox9^+^EpCAM^−^CD24^+^ cells remarkably downregulate hepatocyte markers and express some cholangiocyte markers

**Figure 5:**
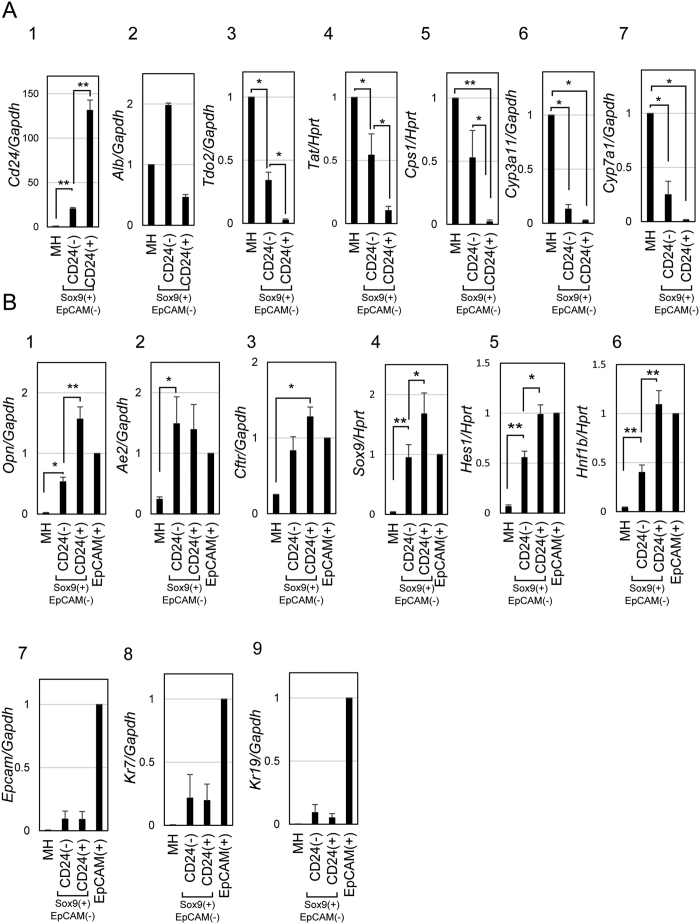
Sox9^+^EpCAM^−^CD24^+^ cells reduce hepatocyte markers and partly acquire cholangiocyte-type gene expression. (**A**) Expression of hepatocyte markers in MHs, Sox9^+^EpCAM^−^CD24^−^, Sox9^+^EpCAM^−^CD24^+^ cells isolated from DDC-injured livers. Hepatocyte markers are reduced in Sox9^+^EpCAM^−^CD24^−^ as compared with MHs, and further downregulated in Sox9^+^EpCAM^−^CD24^+^ cells. Cells were isolated from livers of Sox9-EGFP mice fed with DDC-diet for 2 weeks. Cell isolation was performed 3 times, independently. Error bars represent SEM. The levels of gene expression are presented as relative values against the expression level in MHs isolated from DDC-fed mice. Error bars represent SEM. * and **indicate P < 0.05 and P < 0.01, respectively. (**B**) Expression of cholangiocyte markers in MHs, Sox9^+^EpCAM^−^CD24^−^, Sox9^+^EpCAM^−^CD24^+^, and EpCAM^+^ cells isolated from DDC-injured livers. *Opn* and *Ae2, Cftr, Sox9, Hes1, and Hnf1b* are induced in Sox9^+^EpCAM-CD24^−^ cells. *Opn, Sox9, Hes1, and Hnf1b* are further upregulated in Sox9^+^EpCAM-CD24^+^ cells. On the other hand, *Epcam, Ck7*, and *Ck19* are barely expressed in both fractions. Cells were isolated from livers of Sox9-EGFP mice fed with DDC-diet for 2 weeks. Cell isolation was performed 3 times, independently. Error bars represent SEM. The levels of gene expression are presented as relative values against the expression level in EpCAM^+^ cells isolated from DDC-fed mice. Error bars represent SEM. * and **indicate P < 0.05 and P < 0.01, respectively.

**Figure 6:**
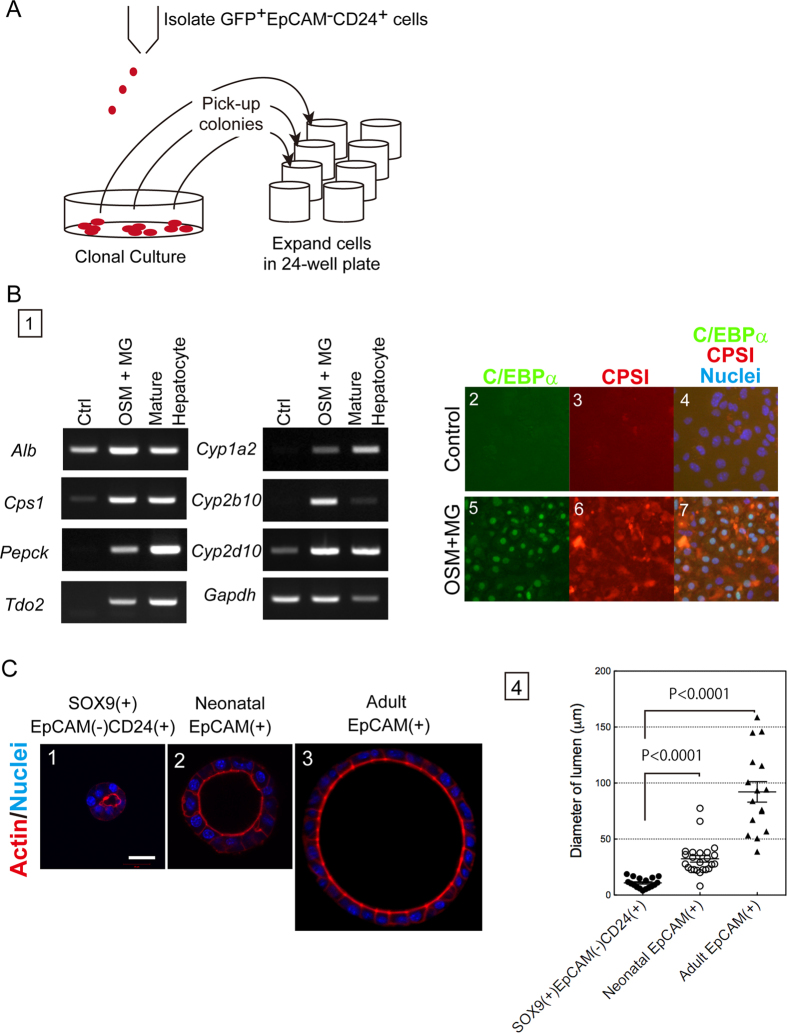
Differentiation potential of Sox9^+^EpCAM-CD24^+^ cells. (**A**) Establishment of cell lines derived from Sox9^+^EpCAM^−^CD24^+^ cells. Sox9^+^EpCAM^−^CD24^+^ cells were isolated from Sox9-EGFP mice fed with DDC diet for 2 weeks. After 1 month of clonal culture, colonies were transferred to wells of a 24-well plate coated with laminin 111. Expanded clones were used for following experiments. (**B**) A hepatocyte progenitor cell line differentiates into hepatocytes. In the presence of OSM and Matrigel, *Alb* expression is increased, whereas metabolic enzymes including *Cps1, Pepck*, and *Tdo2* are induced in Sox9^+^EpCAM^−^CD24^+^ cells (**panel 1**). Expression of C/EBPα (green) and CPSI (red) are detected at protein level (**panels 2~7**). A bar represents 50 μm. (**C**) 3D culture of Sox9^+^EpCAM-CD24^+^ and EpCAM^+^ cells. Sox9^+^EpCAM-CD24^+^ cells form cysts with a tiny lumen (**panel 1**). In the same condition, EpCAM^+^ cells derived from neonate and adult livers form large cysts (**panels 2&3**). A scale bar represents 20 μm. Quantitative analysis indicates cysts derived from Sox9^+^EpCAM-CD24^+^ cells are significantly smaller than those from neonatal and adult EpCAM^+^ cells (**panel 4**). Cells were plated on gel containing Matrigel and type I collagen and them overlaid with 5% Matrigel. At 7 days of culture, images were collected with a digital camera set on a microscope and used to measure the diameter of the central lumen. 3D culture was repeated 3 times and a representative result is shown in this figure.

**Figure 7:**
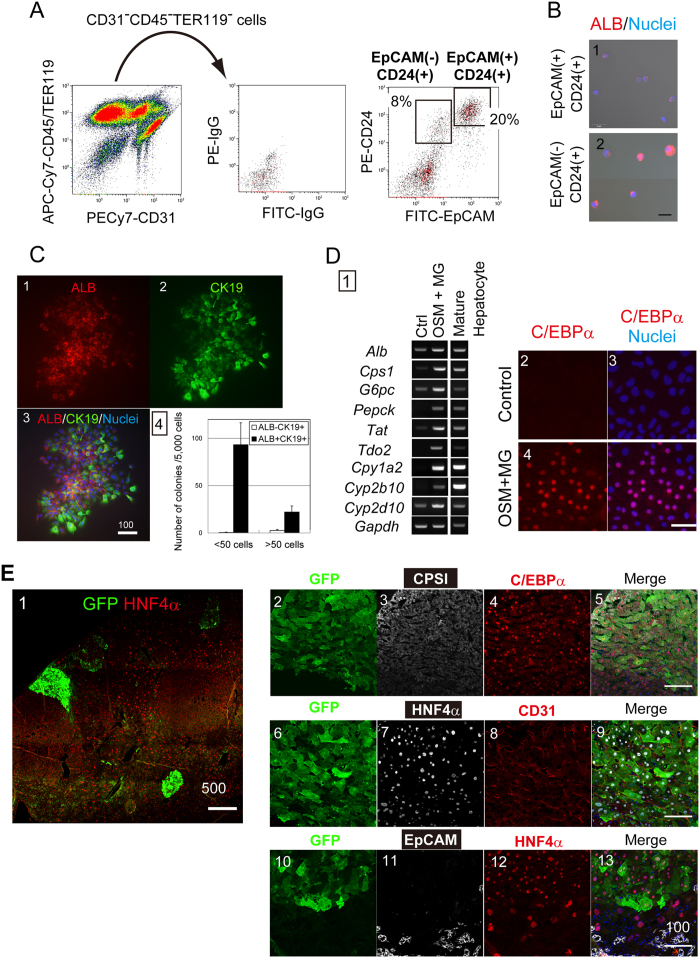
EpCAM^−^CD24^+^ biphenotypic hepatocytes are engrafted as MHs in the recipient liver. (**A**) Isolation of hepatocyte progenitors from the wild type mice fed with DDC-diet. About 8 and 20% of CD31^−^CD45^−^TER119^−^ are EpCAM^−^CD24^+^ and EpCAM^+^CD24^+^ cells, respectively. Mice were fed with DDC-diet for 2 weeks and used for cell isolation. (**B**) Expression of ALB in EpCAM^−^CD24^+^ and EpCAM^+^CD24^+^ cells. EpCAM^−^CD24^+^ but not EpCAM^+^CD24^+^ cells express ALB (red). Smear samples were prepared with a Cytospin and stained with anti-mouse ALB antibody. Nuclei were counterstained with Hoechst 33326. A bar represents 20 μm. (**C**) EpCAM^−^CD24^+^ cells form colonies containing ALB(+) hepatocytes. A typical colony derived from EpCAM^−^CD24^+^ cells is shown (**panels 1~3**). The size of colony varies but most of colonies contain ALB^+^ cells (**black bars in panel 4**). EpCAM^−^CD24^+^ cells were isolated from the wild type mice fed with DDC-diet for 2 weeks and clonally cultured for 7 days. Colonies were stained with anti-ALB, anti-CK19 antibodies, and Hoechst 33326. (**D**) EpCAM^−^CD24^+^ cells differentiate to MHs *in vitro*. EpCAM^−^CD24^+^ cells express hepatocyte markers in the presence of OSM and MG (Panel 1). C/EBPα is induced at protein level (**panels 2~5**). A bar represents 50 μm. (**E**) GFP^+^ donor cells are engrafted as hepatocytes in the recipient liver. GFP^+^ cells forming foci are observed in the recipient liver (**panel 1**). GFP^+^ donor cells are C/EBPα and CPSI, indicating that they differentiate to MHs (**panels 2~5**). Furthermore, CD31^+^ sinusoidal endothelial cells clearly form a network inside the GFP^+^ foci, indicating that donor-derived MHs are integrated into recipient liver tissue (**panels 6~9**). On the other hand, GFP^+^EpCAM^+^ cells are not observed in the recipient liver (**panes 10~13**). Bars represent 100 μm.

To further clarify the cellular characteristics of CD24^+^ cells in Sox9^+^EpCAM^−^ biphenotypic hepatocytes, we examined expression of hepatocyte and cholangiocyte markers in CD24^−^ and CD24^+^ fractions of Sox9^+^EpCAM^−^ as well as MHs. First, we confirmed the purity of CD24^−^ and CD24^+^ fractions by examining *Cd24* expression (Fig. 5A-1). *Albumin (Alb*) was clearly expressed in all three fractions (Fig. 5A-2), whereas metabolic enzymes including *tyrosine aminotransferase (Tat*) and *tryptophane oxygenase (Tdo2*) decreased in Sox9^+^EpCAM^−^CD24^−^ cells as compared with MHs and were further reduced in CD24^+^ ones (Figs 5A-3 & 4). *Carbamoylphosphate synthetase 1 (Cps1*) was slightly downregulated in CD24^−^ cells, whereas it was significantly downregulated in CD24^+^ cells (Fig. 5A-5). *Cytochrome P450s (Cyps*) were remarkably downregulated in both fractions as compared with MHs (Fig. 5A-6 & 7). Among cholangiocyte markers, *Opn, anion exchanger 2 (Ae2*), *Sox9, hairy enhancer of slit 1 (Hes1*), and *hepatocyte nuclear factor 1 β (Hnf1b*) were upregulated in CD24^−^ cells as compared with MHs (Fig. 5B-1). *Cystic fibrosis transmembrane conductance regulator (Cftr*) was upregulated in CD24^+^ cells (Fig. 5B-3). Expressions of *Opn, Sox9, Hes1*, and *Hnf1b* were augmented in CD24^+^ than CD24^−^ cells. On the other hand, *Epcam, cytokeratin 7 (Kr7*) and *Kr19* were not upregulated in either fractions (Fig. 5B-7~9). Collectively, hepatocyte markers and part of cholangiocyte markers were downregulated and upregulated, respectively, in CD24^+^ cells more prominently than in CD24^−^ ones.

### Sox9^+^EpCAM^−^CD24^+^ biphenotypic hepatocytes differentiate into mature hepatocytes *in vitro*

Partial acquisition of cholangiocyte-type gene expression and formation of colonies consisting of both ALB^+^ and CK19^+^ cells in clonal culture suggested that Sox9^+^EpCAM^−^CD24^+^ cells might acquire cellular characteristics as intermediate cells of hepatocyte-to-cholangiocyte conversion or bipotential liver progenitors. We first examined differentiation potential of Sox9^+^EpCAM^−^CD24^−^ and Sox9^+^EpCAM^−^CD24^+^ cells freshly isolated from DDC-injured liver in cultures inducing hepatocyte and cholangiocyte differentiation. Although CD24^−^ cells expressed hepatocyte markers more than CD24^+^ ones at the time of cell isolation (Fig. 5), their expression had become similar level in both cell populations during culture for a week before inducing hepatocyte differentiation (Fig. S3A). Both CD24^−^ and CD24^+^ cells differentiated to express hepatocyte markers in the presence of oncostatin M (OSM) and Matrigel (Fig. S3A). In three dimensional (3D) culture, CD24^+^ cells formed small cysts slightly more frequently that CD24^−^ ones, though the difference in the capability forming the cyst structure was not substantially different between these two populations (Fig. S3C). To further clarify differentiation potential of Sox9^+^EpCAM^−^CD24^+^ cells, we clonally cultured them and established 3 cell lines (Figs 6A and S4). They maintained the status of Sox9^+^EpCAM^−^CD24^+^. In the following part of this section, we demonstrate representative data of Clone No. 3 in culture inducing either hepatocyte or cholangiocyte differentiation. We first performed a culture inducing hepatocyte differentiation with oncostatin M (OSM) treatment followed by Matrigel overlay. In the presence of OSM and Matrigel, Sox9^+^EpCAM^−^CD24^+^ cells expressed metabolic enzymes including *Cps1, Pepck*, and *Tdo2* and cytochrome P450s including *Cyp1a2, 2b10*, and *2d10* at mRNA level (Fig. 6B-1). They expressed C/EBPα and CPSI at protein level (Fig. 6B-2~7). Next, we performed three dimensional (3D) culture, in which bipotential liver progenitors and cholangiocytes form cysts, spherical structures with a central lumen. For this experiment, we used a bipotential liver progenitor cell line derived from a neonatal EpCAM^+^ cell^21^ and adult EpCAM^+^ cholangiocytes that were expanded on type I collagen gel before 3D culture as positive controls forming the cyst structures (Fig. 6C-2 & 3). Although Sox9^+^EpCAM^−^CD24^+^ cells occasionally formed cysts in this culture condition, the size of the central lumen was much smaller than bipotential liver progenitors and adult cholangiocytes (Fig. 6C-1 & 4). Efficient and limited differentiation into MHs and cholangiocyte-like cells, respectively, are common feature of 3 clones derived from Sox9^+^EpCAM^−^CD24^+^ cells. Collectively, in spite of partial acquisition of cholangiocyte-type gene expression profile and the ability forming colonies containing both ALB^+^ and CK19^+^ cells, Sox9^+^EpCAM^−^CD24^+^ cells are hepatocyte progenitor cells that efficiently re-differentiate into MHs but very limitedly into cholangiocyte-like cells.

### EpCAM^−^CD24^+^ biphenotypic hepatocytes are engrafted as MHs in the recipient liver

In order to examine differentiation potential of CD24^+^ biphenotypic hepatocytes *in vivo*, we transplanted them into nude mice after intraperitoneal injections of retrorsine and 70% partial hepatectomy (PHx)^10^. For detecting donor cells in the recipient liver tissue, we usually label donor cells with GFP by using a lentivirus vector and purify GFP^+^ cells by FACS^22^. However, because Sox9^+^EpCAM^−^CD24^+^ cells isolated from Sox9-EGFP mice express GFP from the transgene, we cannot isolate cells introduced with lentivirus vector by using GFP as a marker. To overcome this obstacle, we tried to isolate a cell population identical to Sox9^+^EpCAM^−^CD24^+^ from the wild type mice fed with DDC-diet.

FACS analysis identified EpCAM^−^CD24^+^ cells in CD31^−^CD45^−^TER119^−^ fraction of the wild type mice fed with DDC-diet. Then we isolated those EpCAM^−^CD24^+^ cells and examined their cellular characteristics. For cell isolation, we carefully set FACS gating to avoid contamination of MHs (Fig. S5). EpCAM^−^CD24^+^ cells were positive for ALB, HNF4α and SOX9 (Figs 7B and S6B). The size of EpCAM^−^CD24^+^ cells was significantly smaller and large than MHs and EpCAM^+^CD24^+^ cholangiocytes, respectively (Fig. S6D). EpCAM^−^CD24^+^ cells showed colony-forming capability (Fig. 7C). These results indicated that CD24^+^ biphenotypic hepatocytes were successfully enriched in this fraction. We labeled them with GFP, purified GFP^+^ cells by FACS, and then expanded to acquire the enough number of cells for transplantation. Before transplantation, we confirmed that EpCAM^−^CD24^+^ cells kept potential to differentiate into MHs (Fig. 7D). The donor cells were injected into the spleen of the retrorsine/PHx model of nude mouse. Three months after transplantation, we found that donor cells formed GFP^+^ foci in the recipient liver (Fig. 7E-1). The repopulation efficiency of EpCAM(−)CD24(+) cells was about 1.5%. GFP^+^ cells were positive for C/EBPα and CPSI, indicating that they differentiated into MHs (Fig. 7E-2~5). Furthermore, CD31^+^ sinusoidal endothelial cells penetrated into the GFP^+^ foci, indicating that the donor cells were engrafted into hepatic cords in recipient liver tissue (Fig. 7E-6~9). On the other hand, we could not find GFP^+^EpCAM^+^ cholangiocyte-like cells (Fig. 7E-10~13). We also examined expression of OPN and SOX9 in recipient liver tissue and found that GFP^+^ cells were negative for both cholangiocyte markers. Taken together, Sox9^+^EpCAM^−^CD24^+^ cells maintain potential to differentiate into MHs for functioning as the reservoir of new hepatocytes for tissue regeneration.

## Discussion

In this study, we demonstrate that Sox9^+^EpCAM^−^ biphenotypic hepatocytes induced in chronically injured livers consist of at least two distinctive population; CD24^−^ and CD24^+^ cells. Clonal proliferative capability mostly attributes to CD24^+^ fraction of Sox9^+^EpCAM^−^ biphenotypic hepatocytes. Although expressions of hepatocytic genes are prominently downregulated in Sox9^+^EpCAM^−^ CD24^+^ cells, they still maintain potential to re-differentiate into MHs *in vitro* and *in vivo*. On the other hand, they have limited potential to differentiate into cholangiocyte-like cells. Such cellular characteristics may be important for efficiently supplying new MHs during regeneration and/or protecting liver tissue from chronic injuries.

It has been recognized that mature epithelial cells, which have been considered to be terminally differentiated, show lineage plasticity upon severe injuries in epithelial organs including the liver, lung, and stomach^23^,^24^,^25^,^26^. However, it remains unknown whether de-differentiation of mature epithelial cells results in producing lineage-committed progenitors, multi-potent stem cells, or different lineage cells. In severely injured trachea, basal cells convert to pulmonary stem cells^25^. On the other hand, in mammary glands, the conversion from basal cells into myoepithelial ones is quite inefficient^27^. It has been reported that hepatocytes can convert to cholangiocytes and vice versa in certain types of chronic liver injuries^15^,^26^. However, as we show in this study, MHs remarkably lose their original characteristics at Sox9^+^EpCAM^−^CD24^+^ status in chronically injured livers but not acquire a potential to differentiate to cholangiocyte-like cells. This properly explains the result shown in previous papers that hepatocyte to cholangiocyte conversion is an inefficient process^16^,^28^. Since Sox9^+^ hepatocytes can re-differentiate into hepatocytes *in vitro* and *in vivo*^19^,^29^, it is temptingly speculated that the complete lineage conversion from hepatocyte to cholangiocyte is suppressed in biphenotypic hepatocytes to maintain potential differentiating into MHs for securing efficient supply of new MHs during tissue regeneration. It may be interesting to further investigate that the similar limitation of lineage conversion is applicable to other tissues and organs.

It was previously reported that LPCs are enriched in CD45^−^TER119^−^CD24^+^ fraction isolated from healthy and DDC-injured livers and that CD24^+^ cells differentiate into hepatocytes in recipient livers upon transplantation^30^. However, according to our results, CD45^−^TER119^−^CD24^+^ cells may contain both EpCAM^+^ LPCs and Sox9^+^EpCAM^−^ biphenotypic hepatocytes. Given that Sox9^+^EpCAM^−^ cells efficiently differentiate to hepatocytes, it may be plausible that induced biphenotypic hepatocytes as well as LPCs enriched in CD45^−^TER119^−^CD24^+^ fraction differentiate into hepatocytes.

The genetic lineage tracing using Mx1Cre:ROSA26 or AAV8-TBG-Cre:ROSA26 system indicates that Sox9^+^EpCAM^−^ biphenotypic cells are derived from MHs. However, we have not been able to address whether a specific population of MHs de-differentiates to become biphenotypic cells. A recent report demonstrated that Sox9^+^ “hybrid” hepatocytes exist in the periportal region of a healthy liver and contribute to liver regeneration^31^. These “hybrid” hepatocytes may just expand upon chronic liver injuries and constitute a compartment of biphenotypic hepatocytes. However, Sox9^+^CD24^+^ biphenotypic hepatocytes possessing progenitor properties emerge only when liver injuries have progressed. Thus, even if periportal “hybrid hepatocytes” function as progenitors for liver regeneration, further de-differentiation of hepatocytes including the transition from CD24^−^ to CD24^+^ status must be induced for driving liver regeneration depending on hepatocyte progenitors.

In this study, using mouse chronic liver injury models, we demonstrate that Sox9^+^ biphenotypic hepatocytes emerged in chronically injured livers consist of CD24^−^ and CD24^+^ cells. At CD24^+^ status, biphenotypic hepatocytes acquire strong proliferative capability, whereas they maintain potential re-differentiating into MHs. It has been recognized that mature epithelial cells can de-differentiate or convert into progenitors, stem cells, and other types of cells facing severe tissue injuries. If we can control emergence of stem/progenitor-like cells from mature epithelial cells and induce their differentiation into any specific lineages *in situ*, it would be expected to promote the recovery from severe tissue damages without organ or cell transplantation. However, there must be molecular mechanisms determining the degree of de-differentiation and lineage conversion in each tissue and organ. Therefore, to use such lineage plasticity for regenerative medicine, it is crucial to know how de-differentiation and lineage conversion proceed and are controlled in target tissues and organs.

## Materials and Methods

### Extracellular matrix, growth factors and chemicals

Growth factor-reduced Matrigel^®^ (MG) and purified laminin-111 were from BD Biosciences (Bedford, MA). Collagen type IPC was purchased from Koken Co. Ltd (Tokyo, Japan). Epidermal growth factor (EGF), hepatocyte growth factor (HGF), and OSM were purchased from R&D systems (Minneapolis, MN).

### Mice

C57BL6 mice were purchased from Sankyo Labo Service Corporation, Inc. (Tokyo, Japan). Sox9-EGFP mice^32^ were used to isolate cells expressing Sox9 as GFP^+^ cells. Chronic liver injuries associated with ductular reaction were induced in Sox9-EGFP mice by feeding them with DDC-containing diet. Common bile duct of a mouse was tied off by a ligature to induce cholestasis. All animal experiments were approved by the Sapporo Medical University Institutional Animal Care and Use Committee and were carried out under the institutional guidelines for ethical animal use.

### Immunohistochemistry

Liver tissues from DDC-injured livers and BDL-injured ones were fixed in Zamboni solution and PBS containing 4% paraformaldehyde (PFA) at 4 °C overnight, respectively. After washing in PBS and then PBS containing 20% sucrose, they were embedded in OCT compound. Thin frozen sections were prepared with a Cryostat CM1950 (Leica microsystems, Wetzlar, Germany). After permeabilization with 0.2% Triton X-100 and blocking with Blockace (DS Pharma Biomedical Co., Ltd., Osaka, Japan), liver sections were incubated with primary antibodies listed in Table S2.

### Cell isolation and Microarray analysis

Healthy and DDC-injured livers of Sox9-EGFP mice were digested with a two-step collagenase perfusion method as described previously^19^. MHs were collected by centrifugation at 50 *g *× 1 min and then Percoll gradient centrifugation at 45 *g *× 15 min was performed to eliminate dead cells. Undigested tissue after collagenase perfusion was further incubated in collagenase/hyaluronidase solution and then EpCAM^+^ cholangiocytes were isolated by FACS AriaII (BD biosciences). Biphenotypic hepatocytes were isolated as GFP^+^EpCAM^−^ cells by FACS AriaII. The details of antibodies used for cell isolation are listed in Table S2. Total RNA was isolated from MHs, cholangiocytes, and Sox9^+^EpCAM^−^ biphenotypic hepatocytes. Microarray analysis was performed by Miltenyi Biotech (Galdbach, Germany).

### Colony assay

Five thousands of cells isolated by FACS were plated in 35-mm dish coated with laminin 111. The cells were cultured in DMEM/F-12 medium supplemented with 10% FBS, 10 ng/ml EGF, 10 ng/ml HGF, 1 × 10^−7^ M dexamethasone (Dex, Sigma-Aldrich), 1x ITS (Gibco, Grand Island, NY), and 20 μM Y-27632 (Wako Pure Chemical Industries, Tokyo, Japan). After 7 days of culture, cells were fixed in 4% PFA and stained with rabbit anti-CK19, goat anti-ALB antibodies. Images for samples were acquired on a Nikon X-81 fluorescence microscope.

### PCR

Total RNA was isolated and used for cDNA synthesis. Taqman probes for *Cps1, Grhl2, Hes1, Hnf1b, Hprt, Sox9*, and *Tat* were purchased from Life technologies. Primers used for PCR are shown in Table S3.

### Cell transplantation

Nude mice were used for cell transplantation according to the previous report^10^,^22^. EpCAM^−^CD24^+^ cells labeled with GFP using lentivirus vector were purified by FACSAriaII. Four-week old nude mice were intraperitoneally injected with 60 mg/kg retrorsine (Sigma-Aldrich, St. Louis, MO) every week for 4 times and then 70% of liver was surgically removed at transplantation. GFP^+^ cells (1 × 10^5^ cells) were injected into spleen.

### Statistical analysis

Statistical analyses for colony assay and qPCR were performed on Microsoft excel to acquire standard error of the mean (SEM). Two-tails Student’s t-test was also performed on Microsoft excel. Statistical analysis for the lumen size of cysts were performed on Prism to acquire SEM and P-values of two-tails Student’s t-test.

## Additional Information

**How to cite this article**: Tanimizu, N. *et al*. Progressive induction of hepatocyte progenitor cells in chronically injured liver. *Sci. Rep.* **7**, 39990; doi: 10.1038/srep39990 (2017).

**Publisher's note:** Springer Nature remains neutral with regard to jurisdictional claims in published maps and institutional affiliations.

## Supplementary Material

Supplementary Figures and Tables
