# Supplementary material for: Progressive induction of hepatocyte progenitor cells in chronically injured liver
Source: Sci Rep. 2017 Jan 4;7:39990. doi: 10.1038/srep39990 (PMC5209740; doi:10.1038/srep39990)
Supplement: Supplementary Figures and Tables [file srep39990-s1.pdf]

Progressive induction of hepatocyte progenitor cells in chronically injured liver

†Naoki Tanimizu[1], Norihisa Ichinohe[1], Masahiro Yamamoto[2], Haruhiko Akiyama[3], Yuji Nishikawa[2], and Toshihiro Mitaka[1]

Supplementary Information

Table S1. Ratio of $CD24^+$ hepatocytes and cholangiocytes in $Sox9^+$ cells

			Number of cells / mm^2
DDC	Cholangiocyte (HNF4α^-)	$CD24^-$	63.6± 14.0
		$CD24^+$	285.6± 34.4
	Hepatocyte (HNF4α^+)	$CD24^-$	64.0 ± 18.8
		$CD24^+$	7.2 ± 1.6
BDL	Cholangiocyte (HNF4α^-)	$CD24^-$	56.8 ± 36.4
		$CD24^+$	663.2± 36.4
	Hepatocyte (HNF4α^+)	$CD24^-$	106.4 ± 30.4
		$CD24^+$	14.4 ± 4.0

Liver sections prepared from Sox9-EGFP mice fed with DDC or given BDL treatment were stained with anti-GFP, anti-CD24 and anti-HNF4α antibodies. Sections were prepared from 3 different mice. Three areas were randomly selected from each section and used for counting cells.

Table S2. Primary Antibodies

Antibody	Company	Host animal	Method	Dilution
Albumin	Bethyl laboratory	goat	IF	1:1000
CD16/32	BD Pharmingen	rat	FACS	1:1000
CD24	Biolegend	rat	FACS<br>IF	1:1000<br>1:200
CD31	Biolegend	rat	FACS	1:1000
CD45	BD Pharmingen	rat	FACS	1:1000
Cytokeratin 19	Tanimizu et al. 2003	rabbit	IF	1:2000
EpCAM	BD Pharmingen	rat	IF	1:500
EpCAM<br>(FITC or APC-conjugated)	Biolegend	rat	FACS	1:1000
GFP	MBL	rabbit	IF	1:1000
Grhl2	Sigma-Aldrich	rabbit	IF	1:500
HNF1β	Santa Cruz	rabbit	IF	1:200
HNF4α	SantaCruz<br>Biotechnology Inc.	rabbit	IF	1:200
HNF4α	SantaCruz<br>Biotechnology Inc.	goat	IF	1:200
Sox9	Millipore	rabbit	IF	1:2000
TER119	BD Pharmingen	rat	FACS	1:1000

Table S3. Primers used for PCR

Gene name		Sequence
Albumin	Sense	atgagattctgacccagtgttg
	Antisense	ttctccttcacaccatcaagc
Ck7	Sense	accctcaacaacaaattcgcctcc
	Antisense	tgctcttggctgacttctgttcct
Ck19	Sense	agattgagagagaacacgccttgc
	Antisense	tcaggctctcaatctgcatctcca
CPSI	Sense	tgggatcttgaccgtttcc
	Antisense	accaatggccatgacctc
Cyp3a2	Sense	ccctgcccttcagtggtaca
	Antisense	gaagagccgagtcatggaag
Cyp2b10	Sense	gttgagccaaccttcaaggaa
	Antisense	aagagctcaaacatctggctg
Cyp2d10	Sense	gatcccaaggtgtggtcctt
	Antisense	gcaggagtatggggaacata
EpCAM	Sense	ctgtcatttgctccaaactggcgt
	Antisense	cgttgcactgcttggctttgaaga
HPRT	Sense	tcctcctcagaccgctttt
	Antisense	cctggttcatcatcgctaatc
PEPCK	Sense	ttgatgcccaaggcaactta
	Antisense	acggccaccaaagatgatac
Smo	Sense	gcaagctcgtgctctggt
	Antisense	gggcatgtagacagcacaca
Sox9	Sense	cagcaagactctgggcaag
	Antisense	atcggggtggtctttcttgt
TAT	Sense	caacaacccgtccaatcc
	Antisense	gacgcattgcctttcagc
Tdo2	Sense	tgagtaaaggtgaacgacgac
	Antisense	acggccaccaaagatgatac

Legends to Supplementary Figures

Fig. S1. Sox9(+) biphenotypic hepatocytes are derived from mature hepatocytes (MH).

A. Mature hepatocytes are selectively labeled with LacZ using ROSA26 mice and AAV8-TBG-Cre. Liver sections of ROSA26 mice injected with AAV8-TBG-Cre were used for X-gal staining and for immunostaining. LacZ(+) cells are positive for HNF4α (**panel 1**) but negative for CK19 (**panel 2**), SOX9 (**panel 3**), F4/80 (**panel 4**), Desmin (**panel 5**), and LYVE-1 (**panel 6**). These results indicate that HNF4α (+) hepatocytes are selectively labeled with LacZ in ROSA26 mice injected with AAV8-TBG-Cre. Bars represent 40 μm.

B. **$SOX9^+$ biphenotypic hepatocytes derived from MHs emerge in DDC-injured liver.** $LacZ^+SOX9^+$ biphenotypic hepatocytes are abundantly observed near portal vein (PV) (**arrowheads in panel 2**). $LacZ^+CK19^+$ cholangiocytes that are derived from MHs are only occasionally observed (**arrowhead in panel 4**). Boxes in panels 1 and 3 are enlarged in panels 2 and 4, respectively. ROSA26 mice injected with AAV8-TBG-Cre were fed with normal diet for 2 months and then with DDC-diet for 1 month. Bars represent 40 μm.

Fig. S2. $CD24^-$ $SOX9^+$ biphenotypic cells shift to $CD24^+$ ones during DDC-feeding.

A. **The ratio of $CD24^-$ and $CD24^+$ cells in $SOX9^+$ biphenotypic cells is altered during DDC-feeding.** Sox9-EGFP mice were fed with DDC-diet. At 1W, 2W, and 3W of DDC-feeding, $CD31^-CD45^-$ cells were analyzed for expression of GFP and EpCAM, and then GFP^+EpCAM^- cells were for CD24. The ratio of $CD24^-$ and $CD24^+$ cells are 65 and 35 % at 1W, 35 and 65 % at 2W, and 20 and 80 % at 3W of DDC-feeding.

B. **$SOX9^+EpCAM^-CD24^+$ cells are derived from $SOX9^+EpCAM^-CD24^-$ cells *in vitro*, but not from $EpCAM^+$ ones.** $SOX9^+EpCAM^+$ and $SOX9^+EpCAM^-CD24^-$ cells were isolated from DDC-injured livers. After 2 weeks of culture, progenies of $SOX9^+EpCAM^+$ cells keep expression of SOX9, EpCAM, and CD24. On the other hand,

SOX9$^+$EpCAM$^-$CD24$^-$ cells acquire CD24 expression and thereby become SOX9$^+$EpCAM$^-$CD24$^+$ cells.

C. CD24$^+$ hepatocytes derived from MHs emerge in DDC-injured liver. LacZ$^+$CD24$^+$ hepatocytes are observed near expanded ductular structures in DDC-injured liver (**arrowheads in panel 3**). Box in panel 2 is enlarged in panel 3. ROSA26 mice injected with AAV8-TBG-Cre were fed with normal diet for 2 months and then with DDC-diet (**panel 2 and 3**) or with normal diet (**panel 1**) for 1 month. Bars represent 50 μm.

Fig. S3. Differentiation potential of CD24$^-$ and CD24$^+$ cell sin SOX9$^+$ biphenotypic cells.

A. **CD24$^-$ and CD24$^+$ cells redifferentiate to hepatocytes *in vitro*.** Both CD24$^-$ and CD24$^+$ cells are induced to upregulate hepatocyte markers including *Cps1*, *Tdo2*, and *Cyps* in the presence of OSM and Matrigel (panel 1). They also express C/EBPα and ALB (panel 2). In this culture condition, EpCAM$^+$ cells did not express hepatocytic markers (panel 1). Cells were isolated from SOX9-EGFP mice fed with DDC-diet for 2W.

B. **CD24$^-$ and CD24$^+$ cells do not differentiate to cholangiocyte-like cells *in vitro*.** Both CD24$^-$ nor CD24$^+$ cells formed small cysts in 3D culture. In this culture condition, EpCAM$^+$ cells form large cysts with the central lumen and express CK19.

Fig. S4. Establishment of cell lines derived from Sox9$^+$EpCAM$^-$CD24$^+$ hepatocyte progenitors.

A. Sox9$^+$EpCAM$^-$CD24$^+$ cells maintain expression of GFP (Sox9) and CD24. FACS data for clone 2 are shown. Progenies of Sox9$^+$EpCAM$^-$CD24$^+$ biphenotypic hepatocytes maintain expression of GFP derived from the transgene in Sox9-EGFP mice and that of CD24 (**panel 2**). In contrast, they are EpCAM$^-$ (**panel 2**). HPPL, a progenitors derived from E14 hepatoblasts isolated from a wild type mouse, used as a negative control for

GFP (**panel 1**). Sox9$^+$EpCAM$^-$CD24$^+$ cells were isolated from Sox9-EGFP mice fed with DDC diet for 2W. After 1 month of clonal culture, colonies were transferred to wells 24-well plate coated with laminin 111.

B. **Proliferation of Sox9$^+$EpCAM$^-$CD24$^+$ cells.** Clone 2 and 3 were replated every 3 or 4 days. At replating, we counted the number of cells and plated 2x10^4 cells in a well of 12-well plate coated with laminin 111.

C. **Sox9$^+$EpCAM$^-$CD24$^+$ cell hepatocyte progenitor cell line differentiates into mature hepatocytes.** Expression of C/EBPα (green) and CPSI (red) are induced in the presence of OSM and MG. A bar represents 50 μm.

D. **Sox9$^+$EpCAM$^-$CD24$^+$ clones form tiny cysts in 3D culture.** Clone 1 and 2 occasionally form cyst structures in 3D culture (**panels 1&2**) in which EpCAM$^+$ cells form large cysts with a central lumen (**panel 3**). The central lumens of cysts derived from clone 1 and 2 are significantly smaller than those from adult EpCAM$^+$ cells (**panel 4**). Adult EpCAM$^+$ cells were expanded on type I collagen gel and then used for 3D culture. Bar represents 100 μm.

Fig. S5. Cell isolation from the wild type mice fed with DDC-diet.

A. **The schematic illustration of cell isolation separating MHs from other cell populations.**

B. **The CD31$^-$CD45$^-$EpCAM$^-$CD24$^+$ fraction does not contain MHs.** The live, singlet cells were used to isolate CD31$^-$CD45$^-$EpCAM$^-$CD24$^+$ cells. MHs are not included in this fraction (**panels 5~8**).

Fig. S6. Comparison among MHs, EpCAM$^-$CD24$^+$, and EpCAM$^+$ cells isolated from DDC-injured liver.

MHs are HNF4α^{+} but SOX9^{-} (**A-1~4**), whereas EpCAM^{-}CD24^{+} cells are HNF4α^{+}SOX9^{+} (**B-1~4**). On the other hand, EpCAM^{+} cells are HNF4α^{-}SOX9^{+} (**C-1~4**). Moreover, MHs are significantly larger than EpCAM^{-}CD24^{+} cells, whereas EpCAM^{-}CD24^{+} are significantly larger than EpCAM^{+} cholangiocytes (**D**). After 2-step collagenase perfusion of DDC-injured liver, MHs were isolated by centrifugation at 50 x g followed by percoll density gradient centrifugation. Cellular fractions eliminated of MHs were used for isolation of EpCAM^{-}CD24^{+} cells and EpCAM^{+} cholangiocytes by FACS. Smear samples were prepared by Cytospin and stained with anti-HNF4a and anti-SOX9 antibodies. Cells in panel 1 are enlarged in **panels 2~4**. Since EpCAM^{-}CD24^{+} cells on the smear sample were very sparse, cells in 4 different areas in **panel B1** are magnified in **panels B2~4**. Bars in **panels A-1**, **B-1 & C-1**, and in **panels A-4, B-4 & C-4** represent 100 and 20 μm, respectively.

Fig. S1

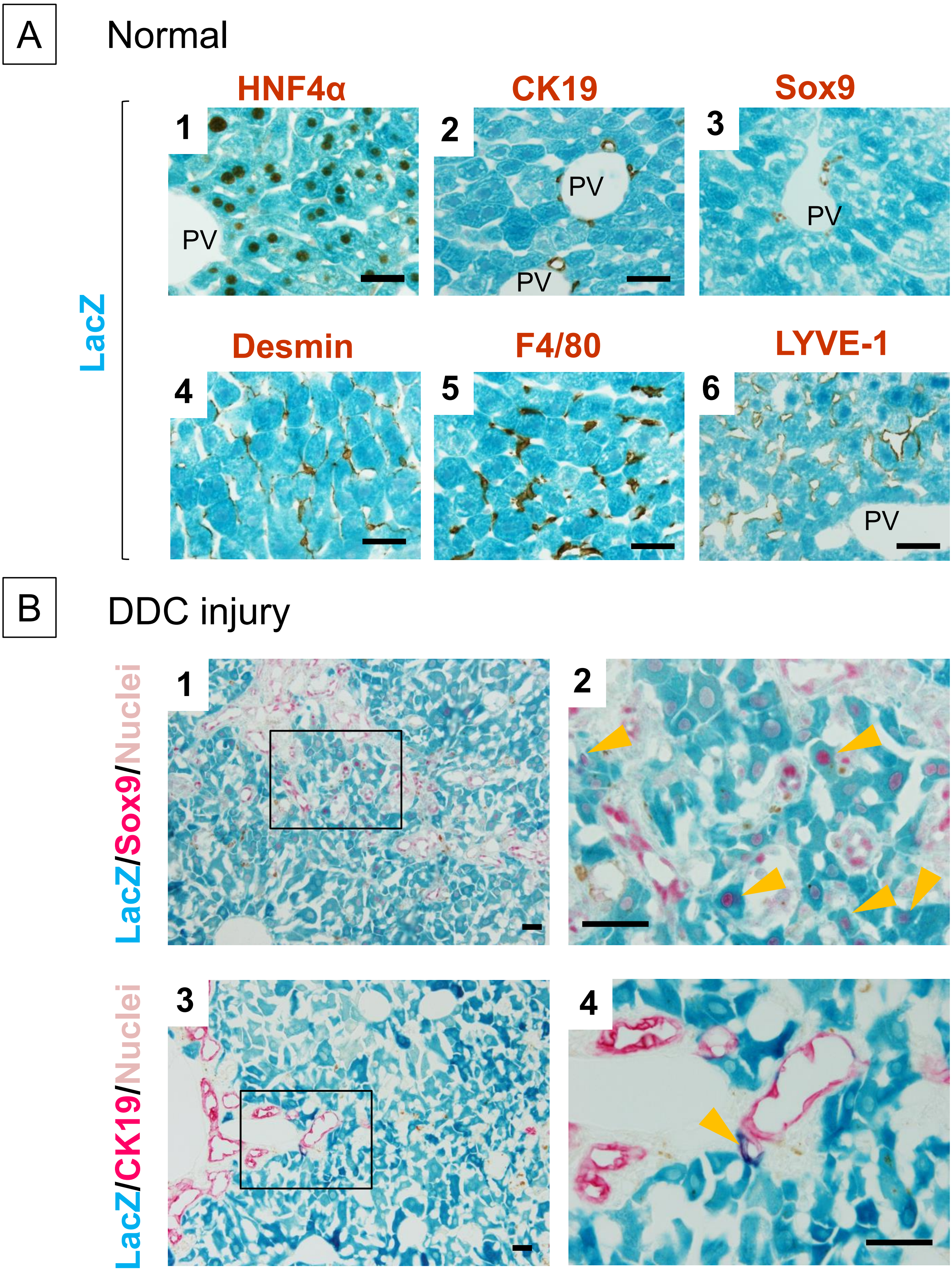
A
Normal
HNF4α
CK19
Sox9
1
2
3
PV
PV
PV
PV
Desmin
F4/80
LYVE-1
4
5
6
PV
LacZ
B
DDC injury
LacZ/Sox9/Nuclei
1
2
LacZ/CK19/Nuclei
3
4

Fig. S2

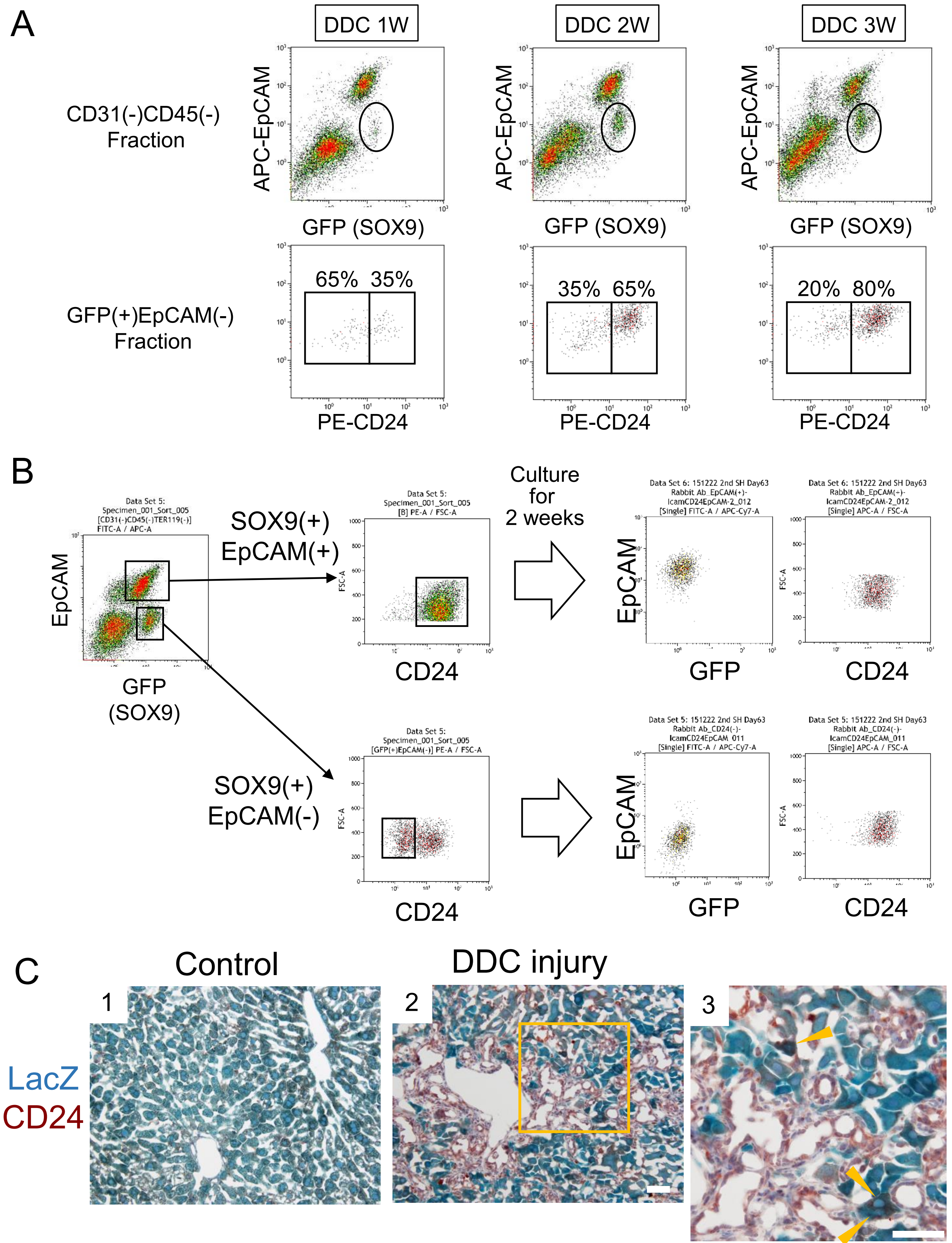
A
DDC 1W
DDC 2W
DDC 3W
CD31(-)CD45(-) Fraction
APC-EpCAM
GFP (SOX9)
GFP(+)EpCAM(-) Fraction
65% 35%
35% 65%
20% 80%
PE-CD24
B
Data Set 5: Specimen_001_Sort_005 [CD31(-)CD45(-)TER119(-)] FITC-A / APC-A
EpCAM
GFP (SOX9)
SOX9(+) EpCAM(+)
SOX9(+) EpCAM(-)
Data Set 5: Specimen_001_Sort_005 [B] PE-A / FSC-A
Data Set 5: Specimen_001_Sort_005 [GFP(+)EpCAM(-)] PE-A / FSC-A
FSC-A
CD24
Culture for 2 weeks
Data Set 6: 151222 2nd SH Day63 Rabbit Ab_EpCAM(+)-IcamCD24EpCAM-2_012 [Single] FITC-A / APC-Cy7-A
Data Set 6: 151222 2nd SH Day63 Rabbit Ab_EpCAM(+)-IcamCD24EpCAM-2_012 [Single] APC-A / FSC-A
Data Set 5: 151222 2nd SH Day63 Rabbit Ab_CD24(-)-IcamCD24EpCAM_011 [Single] FITC-A / APC-Cy7-A
Data Set 5: 151222 2nd SH Day63 Rabbit Ab_CD24(-)-IcamCD24EpCAM_011 [Single] APC-A / FSC-A
GFP
C
Control
DDC injury
1
2
3
LacZ
CD24

Fig. S3

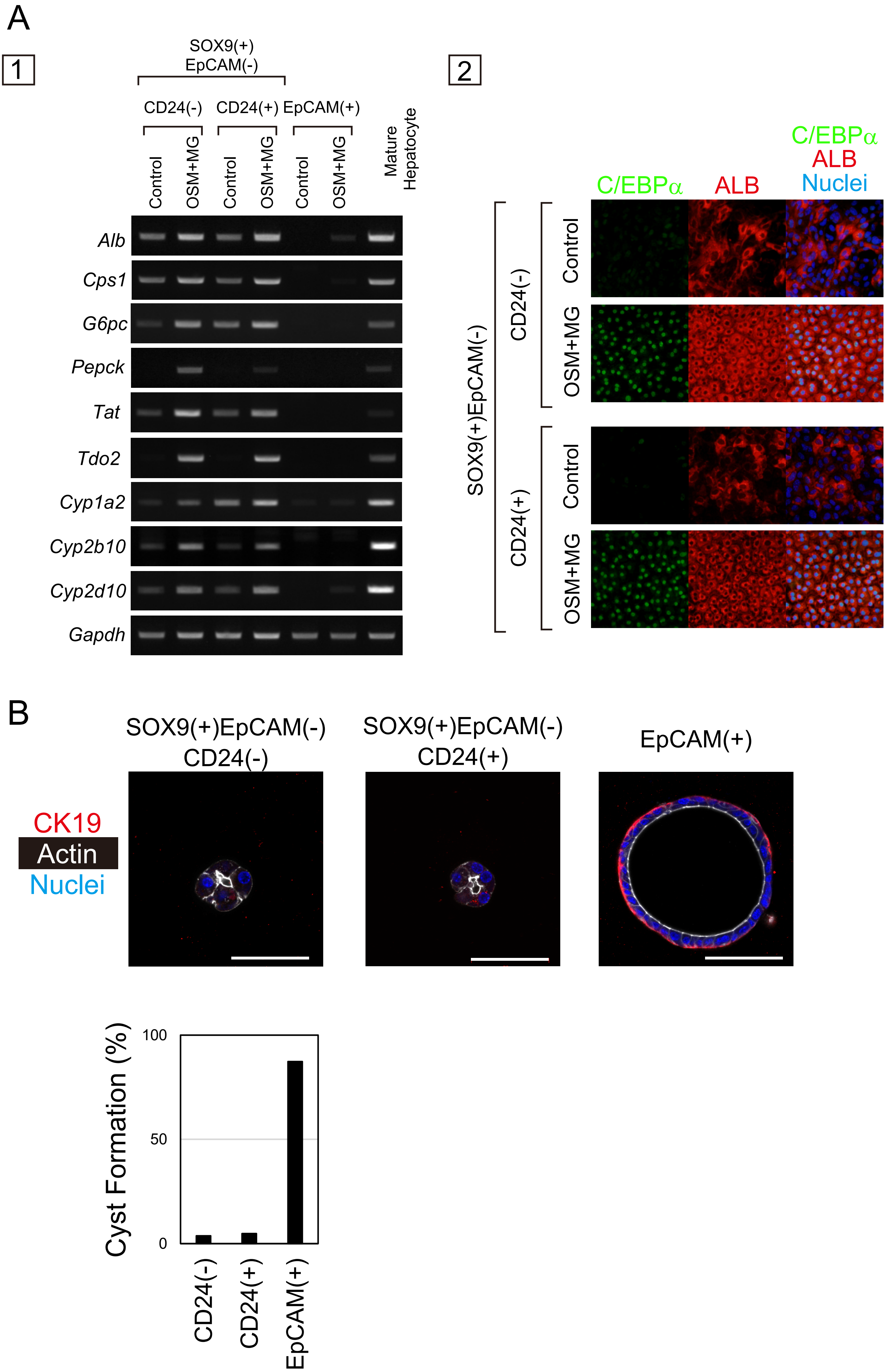
A
1
SOX9(+)
EpCAM(-)
CD24(-)
CD24(+)
EpCAM(+)
Control
OSM+MG
Control
OSM+MG
Control
OSM+MG
Mature Hepatocyte
Alb
Cps1
G6pc
Pepck
Tat
Tdo2
Cyp1a2
Cyp2b10
Cyp2d10
Gapdh
2
C/EBPα
ALB
C/EBPα
ALB
Nuclei
SOX9(+)EpCAM(-)
CD24(-)
Control
OSM+MG
CD24(+)
Control
OSM+MG
B
SOX9(+)EpCAM(-)
CD24(-)
SOX9(+)EpCAM(-)
CD24(+)
EpCAM(+)
CK19
Actin
Nuclei
Cyst Formation (%)
100
50
0
CD24(-)
CD24(+)
EpCAM(+)

Fig. S4

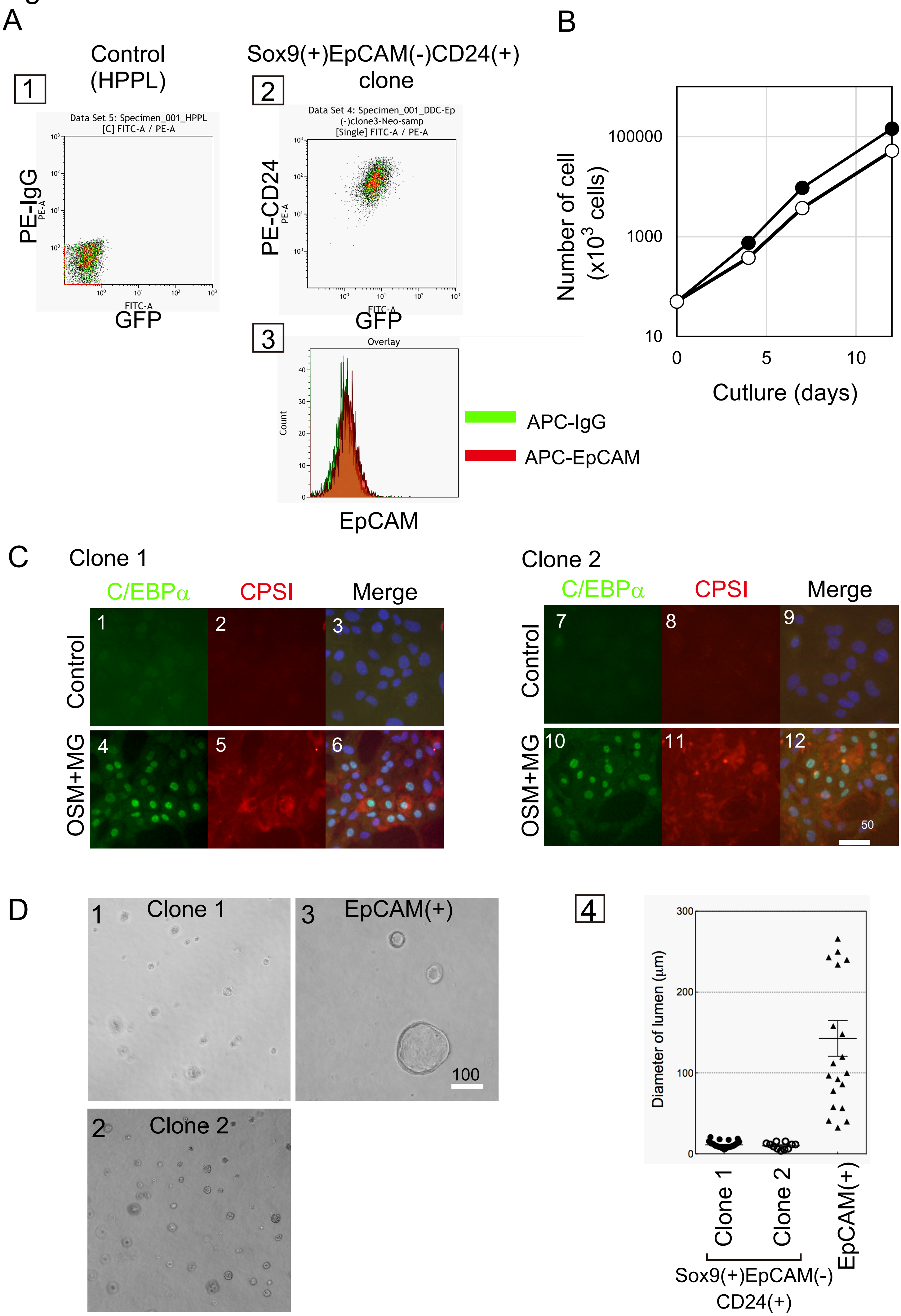

A
Control
(HPPL)
Sox9(+)EpCAM(-)CD24(+)
clone
1
2
3
Data Set 5: Specimen_001_HPPL
[C] FITC-A / PE-A
Data Set 4: Specimen_001_DDC-Ep
(-)clone3-Neo-samp
[Single] FITC-A / PE-A
PE-IgG
PE-CD24
GFP
GFP
Overlay
Count
EpCAM
APC-IgG
APC-EpCAM
B
Number of cell
(x10^3 cells)
100000
1000
10
0
5
10
Cutlure (days)
C
Clone 1
Clone 2
C/EBPα
CPSI
Merge
Control
OSM+MG
50
D
Clone 1
Clone 2
EpCAM(+)
100
4
Diameter of lumen (μm)
300
200
100
0
Clone 1
Clone 2
EpCAM(+)
Sox9(+)EpCAM(-)
CD24(+)

Fig. S5

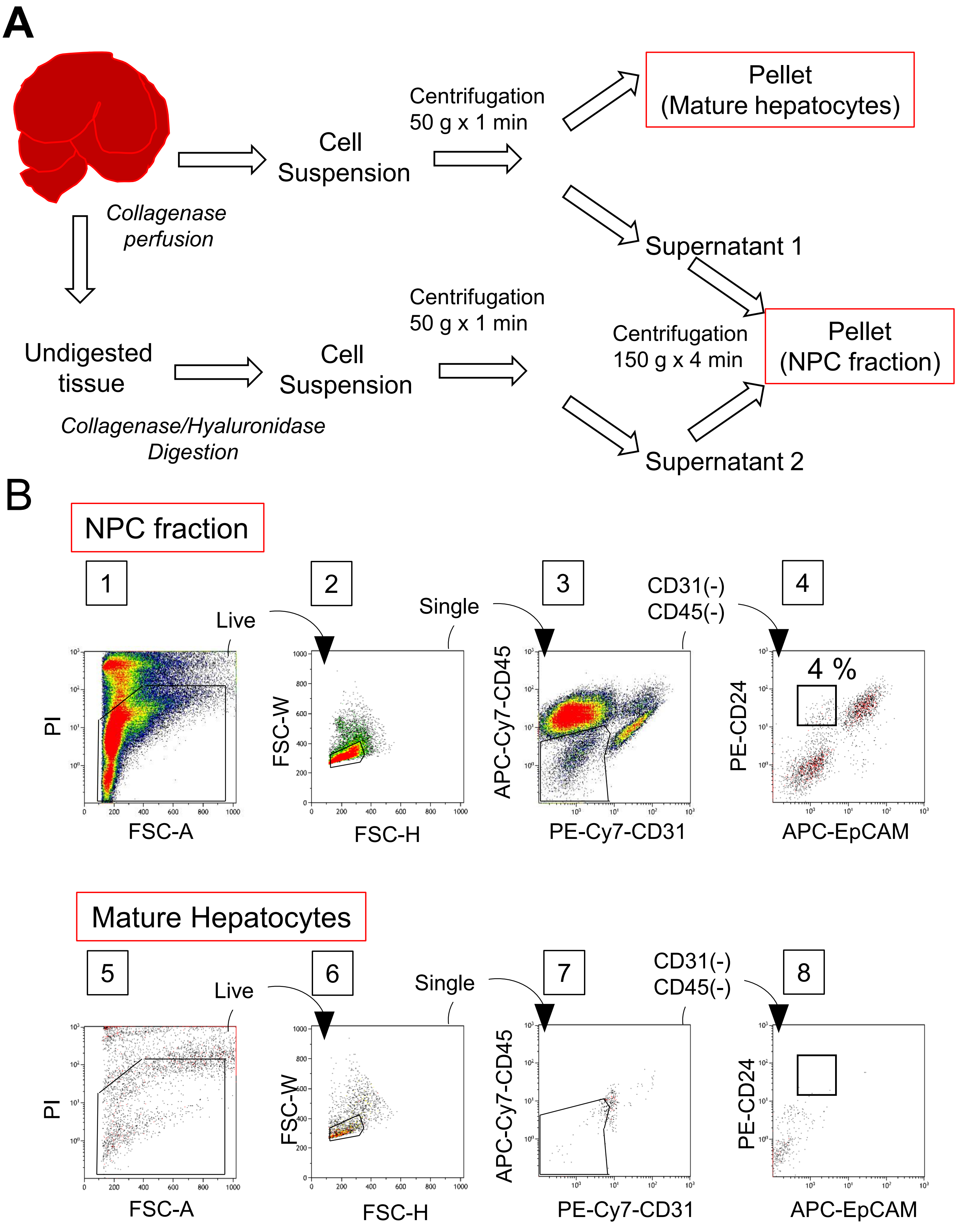
A
Collagenase perfusion
Cell Suspension
Centrifugation 50 g x 1 min
Pellet (Mature hepatocytes)
Supernatant 1
Undigested tissue
Collagenase/Hyaluronidase Digestion
Cell Suspension
Centrifugation 50 g x 1 min
Centrifugation 150 g x 4 min
Pellet (NPC fraction)
Supernatant 2
B
NPC fraction
1
Live
2
Single
3
CD31(-) CD45(-)
4
4 %
PI
FSC-A
FSC-W
FSC-H
APC-Cy7-CD45
PE-Cy7-CD31
PE-CD24
APC-EpCAM
Mature Hepatocytes
5
Live
6
Single
7
CD31(-) CD45(-)
8
PI
FSC-A
FSC-W
FSC-H
APC-Cy7-CD45
PE-Cy7-CD31
PE-CD24
APC-EpCAM

Fig. S6

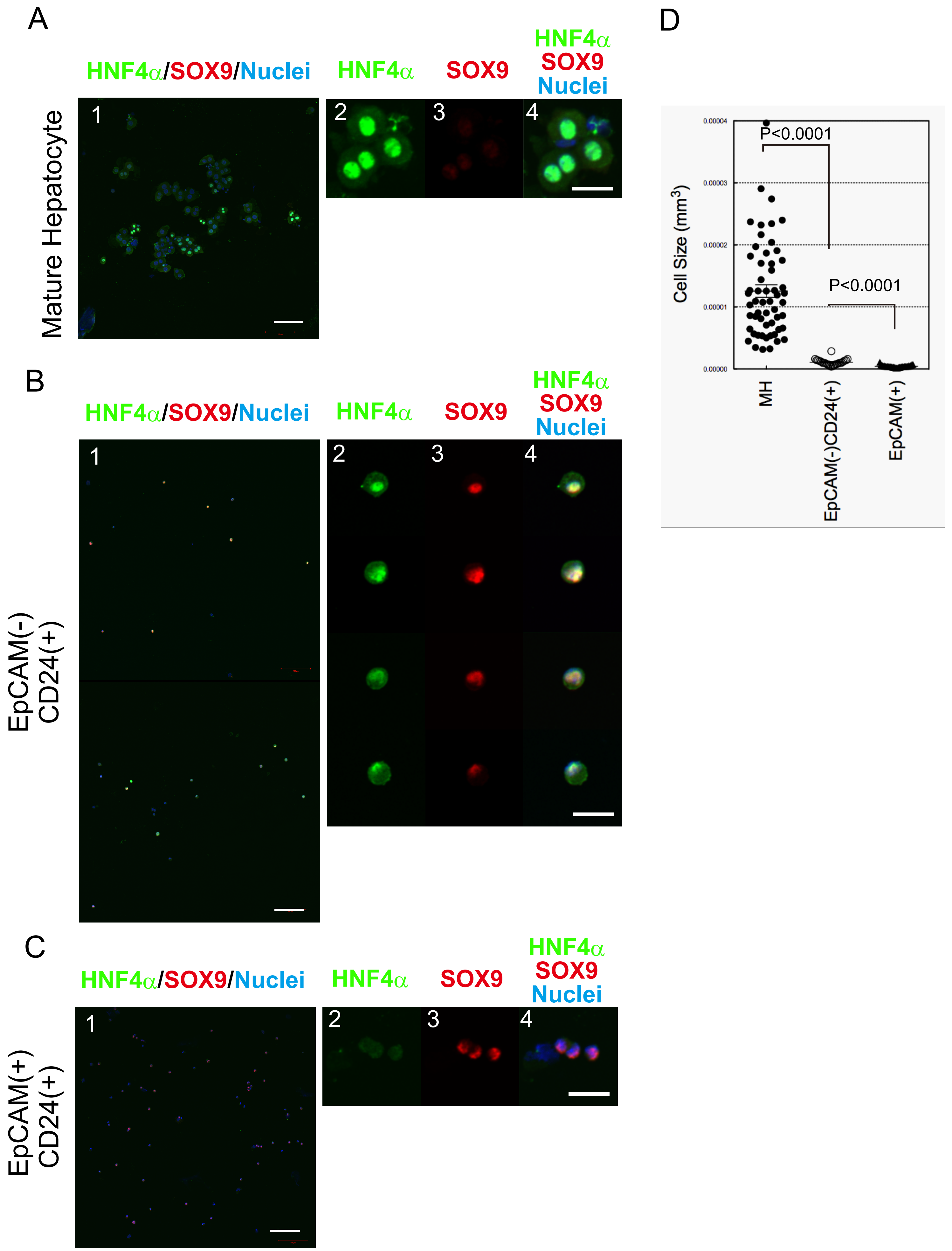

A
HNF4α/SOX9/Nuclei
HNF4α
SOX9
HNF4α
SOX9
Nuclei
Mature Hepatocyte
1
2
3
4
B
HNF4α/SOX9/Nuclei
HNF4α
SOX9
HNF4α
SOX9
Nuclei
EpCAM(-)
CD24(+)
1
2
3
4
C
HNF4α/SOX9/Nuclei
HNF4α
SOX9
HNF4α
SOX9
Nuclei
EpCAM(+)
CD24(+)
1
2
3
4
D
Cell Size (mm3)
0.00004
0.00003
0.00002
0.00001
0.00000
P<0.0001
P<0.0001
MH
EpCAM(-)CD24(+)
EpCAM(+)
